# Social Determinants of Unmet Healthcare Needs: Comparison Between Middle-Aged and Older People

**DOI:** 10.1093/geroni/igaa057.1220

**Published:** 2020-12-16

**Authors:** Sungjae Hong

**Affiliations:** Emory University, Decatur, Georgia, United States

## Abstract

This study aims to 1) examine unmet healthcare needs by age groups and 2) compare the social determinants of unmet healthcare needs between older and middle-aged people in Korea. This study employed a nationally representative dataset of the 2017 Healthcare Service Experience Survey in Korea. Unmet healthcare needs consisted of three categories by healthcare type: 1) clinic visitation, 2) treatment, and 3) medication. Independent variables include demographic, socioeconomic, and health status. Logistic regression models were estimated to reveal the social determinants of unmet healthcare needs of older (age≥65; N=2,178) and middle-aged (age 40~64; N=5,062) people. There was a positive gradient of unmet healthcare needs prevalence by age group, having the highest prevalence among older people (10.8%). While older people living alone were 1.70 times more likely to report any of unmet healthcare needs, there was no significant relationship between the two among middle-aged people. In addition, the effect of chronic disease morbidity on the probability of unmet healthcare needs was stronger among older people than it is among middle-aged people (OR=3.50 and 2.90, respectively). In contrast, the effect of household income was weaker than it is among middle-aged people (OR=1.73 and 2.95, respectively). The gradient of unmet healthcare needs by age group asks gerontologists and public healthcare scholars to focus on older people regarding unmet healthcare needs. Also, the difference between middle-aged and older people on social determinants of unmet healthcare needs implies different psychosocial pathways of unmet healthcare needs between the two age groups.

